# Special Issue: Beneficial Microorganisms for Food Manufacturing—Fermented and Biopreserved Foods and Beverages

**DOI:** 10.3390/microorganisms5040071

**Published:** 2017-11-13

**Authors:** Régine Talon, Monique Zagorec

**Affiliations:** 1 Université Clermont-Auvergne, INRA, MEDIS, F-63000 Clermont-Ferrand, France; 2 Secalim, INRA, LUNAM Université, 44307 Nantes, France

Food fermentation is an ancient technology, disseminated worldwide, which harness microorganisms and their enzymes to improve and diversify the human diet. Fermented foods (vegetables, animal products, beverages) represent 10 to 40% of the global diet, and represent a cultural and gastronomic heritage of high value. The exploration of the microbial communities of these fermented foods has seen renewed interest with the development of metagenomic approaches. Fermentation—either indigenous or after addition of starter cultures—brings many benefits, including (1) enhanced food stability and storage, decreased food losses; (2) enhanced food safety by inhibition of pathogens; (3) improved sensory properties; and (4) improved nutritional value. In many fermented products, the functions underlying all these aspects have to be considered. A better knowledge of microbes and fermentation at the molecular level is required to support and develop the production of sustainable fermented foods with high nutritional characteristics. Investigating the role of starter cultures, as well as that of the indigenous microbiota participating in fermentation, reveals that they are able to guarantee the safety of the products by competing with undesired microorganisms or by producing organic acids—and sometimes other molecules such as H_2_O_2_ or bacteriocins—that have an antagonistic effect towards undesired microorganisms. This safety aspect of the starter cultures led to their use being proposed also in non-fermented products to ensure better microbial safety, or to extend the shelf life of biopreserved food. Such starter cultures become “protective cultures”, and their function is then to contribute to food safety only, without interfering with the sensory aspects of the final product, whether fermented or not.

This issue gathers 13 articles dealing with various aspects of fermented foods and beverages, as well as biopreserved foods. Four of them deal with the fermentation of plants (olive [1], fruit and tea [2,3], gowé [4]) and one concerns goat meat [5]. These articles highlight microbial diversity and its role in sensory and sanitary qualities. Two are dedicated to biopreservation with the aim of controlling pathogens [6] or fungi [7] in food. Three concern well-known starter cultures (*Lactobacillus sakei* [8], *Lactococcus lactis* [9] and *Staphylococcus xylosus* [10]), and explore their potential by a global approach from genome to phenotype. Two articles are related to health, with one focusing on the probiotic properties of dairy propionibacteria [11], and one depicting the nutritional potential of fermented cereals [12]. The last one addresses the regulatory and safety requirements for food cultures [13].
